# Subereamolline A as a Potent Breast Cancer Migration, Invasion and Proliferation Inhibitor and Bioactive Dibrominated Alkaloids from the Red Sea Sponge *Pseudoceratina arabica*

**DOI:** 10.3390/md10112492

**Published:** 2012-11-08

**Authors:** Lamiaa A. Shaala, Diaa T. A. Youssef, Mansour Sulaiman, Fathy A. Behery, Ahmed I. Foudah, Khalid A. El Sayed

**Affiliations:** 1 Natural Products Unit, King Fahd Medical Research Center, King Abdulaziz University, Jeddah 21589, Kingdom of Saudi Arabia; Email: lshalla@kau.edu.sa; 2 Department of Natural Products, Faculty of Pharmacy, King Abdulaziz University, Jeddah 21589, Kingdom of Saudi Arabia; 3 Department of Pharmacology, Faculty of Medicine, King Abdulaziz University, Jeddah 21589, Kingdom of Saudi Arabia; Email: misulaiman@kau.edu.sa; 4 Department of Basic Pharmaceutical Sciences, College of Pharmacy, University of Louisiana at Monroe, Monroe, LA 71201, USA; Email: beheryfa@warhawks.ulm.edu (F.A.B.); foudaha@warhawks.ulm.edu (A.I.F.); elsayed@ulm.edu (K.A.E.S.)

**Keywords:** verongid sponges, *Pseudoceratina arabica*, *Suberea mollis*, subereamolline A, brominated alkaloids, breast cancer, antimigration and anti-invasion assays

## Abstract

A new collection of several Red Sea sponges was investigated for the discovery of potential breast cancer migration inhibitors. Extracts of the Verongid sponges *Pseudoceratina arabica* and *Suberea mollis* were selected. Bioassay-directed fractionation of both sponges, using the wound-healing assay, resulted into the isolation of several new and known brominated alkaloids. Active fractions of the sponge *Pseudoceratina arabica* afforded five new alkaloids, ceratinines A–E (**2**–**6**), together with the known alkaloids moloka’iamine (**1**), hydroxymoloka’iamine (**7**) and moloka’iakitamide (**8**). The active fraction of the sponge *Suberea mollis* afforded the three known alkaloids subereamolline A (**9**), aerothionin (**10**) and homoaerothionin (**11**). Ceratinine B (**3**) possesses an unprecedented 5,7-dibrominated dihydroindole moiety with an epoxy ring on the side chain of a fully substituted aromatic moiety. Ceratinines D (**5**) and E (**6**) possess a terminal formamide moiety at the ethylamine side chain. Subereamolline A (**9**) potently inhibited the migration and invasion of the highly metastatic human breast cancer cells MDA-MB-231 at the nanomolar doses. Subereamolline A and related brominated alkaloids are novel scaffolds appropriate for further future use for the control of metastatic breast cancer.

## 1. Introduction

In the continuation of our interest in exploring the biomedical importance of the secondary metabolites of Verongid sponges [1,2,3,4], and considering the diverse chemical and biological potential of this class as a source for new bioactive entities, we have investigated new collections of two Red Sea Verongid sponges *Pseudoceratina arabica* and *Suberea mollis*. Secondary metabolites derived from members of the genus of *Pseudoceratina* displayed diverse bioactivities including antimicrobial [4,5,6], parasympatholytic [4], HIV inhibition [7], enzyme inhibition [8], cytotoxic [9], and antifouling activity [10,11]. Similar activities including antifungal [12], antibacterial [13,14], cytotoxicity [15,16] and enzyme inhibitory activity [17], were also reported for compounds obtained from members of the genus *Suberea*. Previous investigation of the sponge *P*. *arabica* collected in Sharm El-Sheikh, Egypt, led to the identification of several bioactive compounds including moloka’iamine, hydroxymoloka’iamine, moloka’iakitamide, ceratinophenol A, ceratinamine, 5-bromo-2,3-dihydroxy-6-methoxybenzaldehyde and psammaplysin-A [4]. This study describes the purification and characterization of five new alkaloids; ceratinines A–E (**2**–**6**) together with known alkaloids moloka’iamine (**1**) [18], hydroxymoloka’iamine [4] and moloka’iakitamide [4] from a new collection of the Red Sea sponge *P. arabica *collected in Hurghada at the coast of the Egyptian Red Sea. Furthermore, chromatographic purification of the active antimigratory fraction of a new collection of the sponge *Suberea mollis* resulted in the isolation of the three known alkaloids subereamolline A (**9**) [2], aerothionin (**10**) [12] and homoaerothionin (**11**) [12]. Isolated brominated alkaloids were evaluated for their ability to inhibit the migration, invasion, and viability of the highly metastatic human breast cancer cell line MDA-MB-231.

## 2. Results and Discussion

### 2.1. Structure Elucidation of New Compounds

The FABMS of **2 **displayed three ion peaks at *m/z* 380.9/382.9/384.9 in the ratio of 1:2:1, indicating the dibrominated nature of the molecule. The HRFABMS data of **2** suggested the molecular formula C_12_H_19_^79^Br_2_N_2_O_2_ as established by the molecular ion peak at *m/z* 380.9822 [M + H]^+^, being larger than that of 1 [18] by 30 mass units, suggesting the presence of an additional methoxyl moiety in the molecule. In comparison with compound **1** [18], the absence of the signals of H_2_-7/C-7 in compound **2** and the appearance of new oxygenated methine at δ 4.78 (H-7)/70.4 (CH, C-7) and signal for a methoxyl moiety at δ 3.19 (H_3_-12)/57.6 (C-12) a three-proton singlet at δ 3.19 supported the presence of the methoxyl moiety in **2**. The ^1^H and ^13^C NMR data of **2** (Table 1) together with the HSQC revealed the presence of four methylenes (C-8, C-9, C-10 and C-11), two aromatic methines (C-2 and C-6), one oxygenated aliphatic methine (C-7) and four quaternary carbons (C-1, C-3, C-4 and C-5). The ^1^H and ^13^C NMR data of the basic skeleton of **2** are in good agreement with those reported for moloka’iamine (**1**) [18] suggesting that **2** possesses the moloka’iamine skeleton [18]. Moreover, the ^1^H NMR signal at δ 4.78 (dd, *J *= 9.5 and 3.0 Hz, H-7) together with carbon signal at δ 70.4 (CH, C-7) suggested the oxygenation of C-7. Beside the vicinal COSY correlations with H-7, a geminal correlation between the protons at C-8 was observed with a coupling constant value of 13.0 Hz [4]. The HMBC data confirmed the location of a methoxyl functionality on the ethylamine moiety at C-7 through the cross peaks of H_3_-12/C-7, H-7/C-12, H-7/C-2, C-6, H-7/C-1, H-7/C-8, and H_2_-8/C-1 (Table 1). Thus, compound **2** was assigned as 3-(4-(2-amino-1-methoxyethyl)-2,6-dibromophenoxy)propan-1-amine.

**Table 1:** NMR Spectroscopic Data (Methanol-*d*_4_) for Ceratinines A (**2**) and B (**3**).

	Ceratinine A (2)			Ceratinine B (3)			
Position	δ_C_ (mult.) ^a^	δ_H_ [mult., *J *(Hz)]	HMBC ^b^	δ_C_ (mult.) ^a^	δ_H_ [mult., *J* (Hz)]	δ_H_ [mult., *J* (Hz)] ^c^	HMBC ^b^
1	142.7, C			121.5, C			
2	131.6, CH	7.66, s	1, 3, 4, 5, 7	133.7, C			
3	119.2, C			104.5, C			
4	153.5, C			152.5, C			
5	119.2, C			103.5, C			
6	131.6, CH	7.66, s	1, 3, 4, 5	144.0, C			
7	70.4, CH	4.78, dd (9.5, 3.0)	1, 6, 8	37.2, CH_2_	3.40, t (6.5)	3.05, m	1, 2, 6, 8
8	47.6, CH_2_	2.88, dd (13.0, 3.0) 3.07, dd (13.0, 9.5)	1, 8	49.8, CH_2_	3.16, t (6.5)	2.85, m	1, 7
9	71.6, CH_2_	4.12, t (6.0)	4, 10, 11	72.4, CH	3.75, m	3.70, brs	10, 11
10	29.3, CH_2_	2.20, quin. (6.0)	9, 11	74.6, CH	3.60, dd (10.0, 3.2)	3.35, dd	9, 11
11	38.9, CH_2_	3.27, t (6.0)		73.6, CH	4.00, d (3.2)	4.13, m	10
12	57.6, CH_3_	3.19, s	7	21.3, CH_3_	1.43 ^d^	1.20, s	1, 2, 3
13				20.0, CH_3_	1.43 ^d^	1.15, d (6.5)	9, 10

^a^ Multiplicity of the carbons was assigned from DEPT and HSQC experiment. ^b^ HMBC correlations are from proton(s) stated to the indicated carbon. ^c 1^H NMR data in DMSO-*d*_6_. ^d^ Overlapped signals.

**Figure 1:**
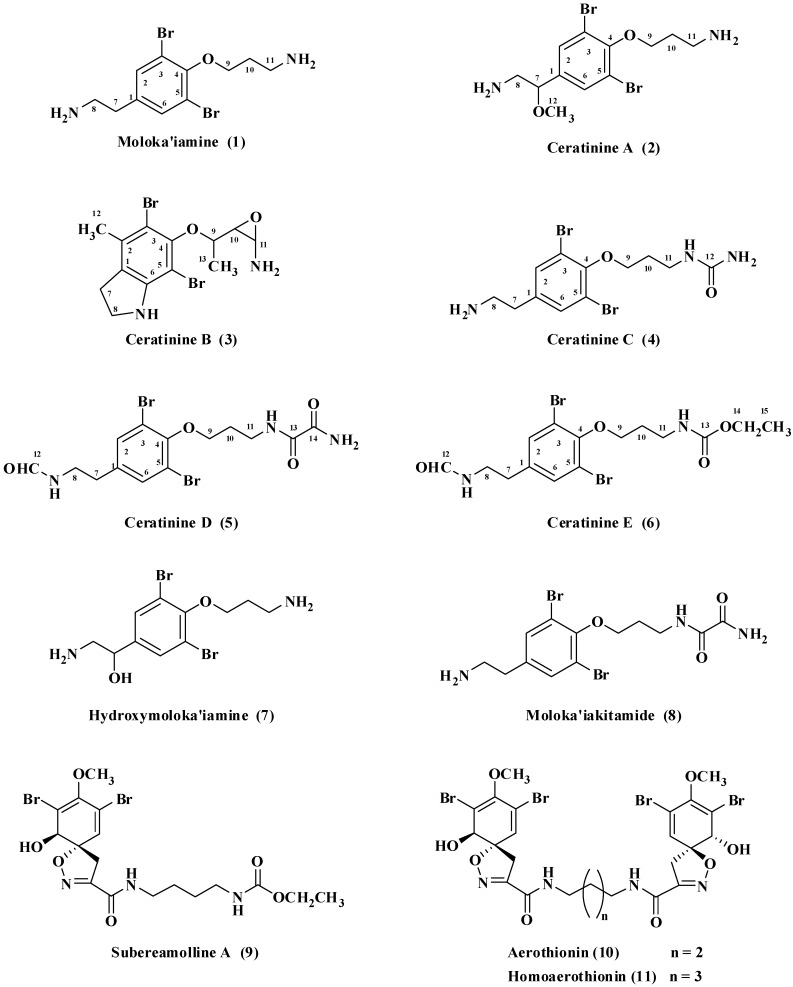
Compounds **1**–**8** isolated from *P. arabica* and compounds **9**–**11** isolated from *S. mollis*.

**Figure 2:**
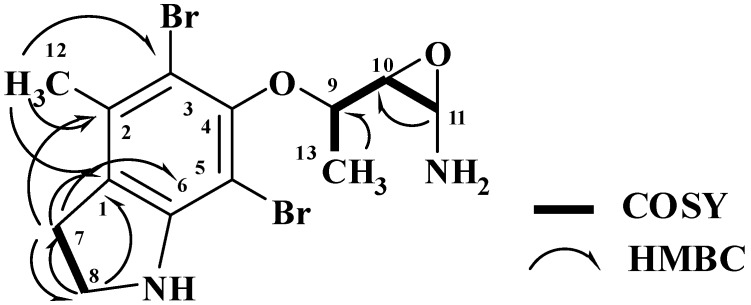
COSY and HMBC Observed for **3**.

**Figure 3:**
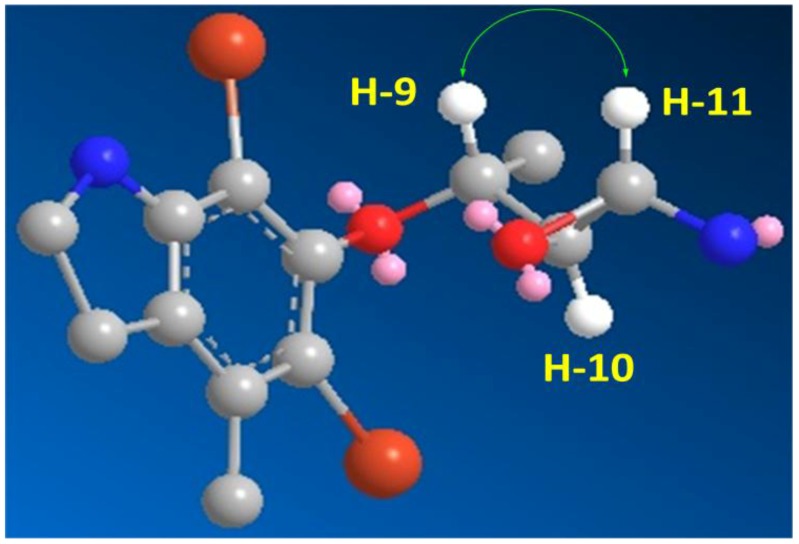
Important H-9 and H-11 NOESY Correlation in **3**.

The HRFABMS analysis of **3** (Figure 1) confirmed the molecular formula C_13_H_17_^79^Br_2_N_2_O_2_ as established from the HRFABMS ion peak at *m/z* 390.9683 corresponding to [M + H]^+^, requiring two degrees of unsaturation more than **2**. Again, the appearance of three ion peaks cluster at *m/z* 390.9/392.9/394.9 in the ratio 1:2:1 suggested the presence of two bromines in **3**. The ^1^H NMR spectra (in CD_3_OD) together with the HSQC revealed the presence of two methyl groups (δ_H/C_ 1.43/20.0 and 1.43/21.3), two methylenes (δ_H/C_ 3.40/37.2 and 3.16/49.8), three oxygenated methines (δ_H/C_ 4.0/73.7, 3.60/74.6 and 3.75/72.4), and six quaternary aromatic carbons between 103.5 and 152.5 ppm, corresponding to the fully substituted aromatic moiety in **3** (Table 1). Interpretation of the COSY experiment revealed the presence of two spin-coupling systems within **3 **(Figure 2). The first spin-coupling system could be traced from H-11 (δ 4.00, d) to H-10 (δ 3.60, dd), which further couples to H-9 (δ 3.75, m). In addition, H-9 showed vicinal coupling to the methyl protons at C-13 (δ 1.43, d), while the second system consists of two triplets of the methylene protons at C-7 (δ 3.40, t, *J* = 6.5 Hz) and C-8 (δ 3.16, t, *J* = 6.5 Hz) (Table 1) of the dihydroindole moiety. Interpretation of HSQC and HMBC (Table 1 and Figure 2) data allowed the assignment of the dihydroindole fragment with a fully substituted benzene ring. The presence of three oxygenated methines (C-9, C-10 and C-11) in the side chain of the molecules and the need for six degrees of unsaturation in **3** support the presence of an epoxy ring at C-10/C-11 to complete the number of unsaturation in the molecule and the structure of **3**. Moreover, the mass fragment ion peaks at *m/z* 374.9 [M − NH_2_ + H]^+^ and 332.9 [M − C_2_H_4_NO + H]^+^ supported the presence of the oxirane functionality at C-10 and 11. HMBC experiment allowed the assignment of the quaternary carbons of the benzene moiety. For example, HMBC cross peaks of H_3_-12/C-3, H_3_-12/C-2, H_3_-12/C-1, H_2_-7/C-2, H_2_-7/C-1, H_2_-7/C-6, H_2_-7/C-8, H_2_-8/C-1 and H_2_-8/C-7 supported the unambiguous assignment of these carbons (Table 1 and Figure 2). The relative stereochemistry at side chain of **3** was assigned from the coupling constant values of H-9, H-10 and H-11 [19,20]. The coupling constant value of 3.2 Hz between H-10 and H-11 reflects their *trans* configuration [19]. Furthermore, the coupling constant of 10.0 Hz between H-9 and H-10 supported their *trans* (*E*)-configuration as well [20]. The strong NOESY correlation between H-9 (d 3.75) and H-10 (d 4.00) supported the assignment. An MM2 ChemBio3D Ultra 11.0 (ChemBioOffice) energy-minimized drawing for compound **3** was created which show the significant NOESY correlation between H-9 and H-11 (Figure 3). Surprisingly, no NOESY correlations were observed between the H_3_-13 and any of the protons on the side chain, which could due to the ring constrain which arise from the substitution on the dihydroindole moiety. 

The available spectroscopic data was not enough for the determination of the absolute configuration of the chiral centers C-9-C-11 of **3 **and therefore, they were left ambiguous. Ceratinine B possesses an unprecedented dibrominated dihydroindole skeleton with an epoxy ring on the side chain and a fully substituted aromatic ring of the indole skeleton. This is the first report of this brominated indole alkaloids in members of the Verongid sponges. Therefore, compound **3** was found to be 3-(1-(5,7-dibromo-4-methylindolin-6-yloxy)ethyl)oxiran-2-amine. 

HRFABMS analysis of **4** (Figure 1) confirmed the molecular formula C_12_H_18_Br_2_N_3_O_2_ as established from the ion peak at *m/z* 393.9772 corresponding to [M + H]^+^, suggesting five degrees of unsaturation, being one more than **2**. Again, the FABMS cluster at *m/z* 393.9, 395.9 and 397.9 [M + H]^+ ^suggested a dibrominated species. A comparison of its NMR data (^1^H, ^13^C, HSQC and HMBC) to that of **2** (Table 2) established the absence of the methoxyl moiety at C-7 of the side in **2**. Furthermore, the appearance of an additional ^13^C NMR signal at δ 160.3 (qC, C-12), was assigned as a carbonyl group of a carbamide moiety attached C-11, completing the degrees of unsaturation and the molecular formula of **4**. The mass fragment ion at *m/z* 350 [M − CONH_2_ + H]^+^ supports the presence of the of the carbamide moiety at one side of the molecule This attachment was secured from HMBC cross-peak of H_2_-11 to C-12 (δ 160.3, Table 2). The assignment of all protonated carbons was securely established from the HSQC data and the HMBC experiment secured the assignment of quaternary carbons (Table 2). Thus, compound **4** was proved to be 1-(3-(4-(2-aminoethyl)-2,6-dibromophenoxy)-propyl)urea.

The molecular formula of **5** (Figure 1) was assigned as C_14_H_18_^79^Br_2_N_3_O_4_ as established from the molecular ion peak at *m/z* 449.9655 [M + H]^+^, suggesting six degrees of unsaturation, two more than **4**. Again, the dibrominated nature of **5** was confirmed from the FABMS cluster at *m/z* 449.9, 451.9 and 453.9. A comparison of the NMR data (Table 2) of **5** (^1^H, ^13^C, HSQC and HMBC) against those of **4** established identical similarity of all signals. Furthermore, the appearance of NMR signals at δ 160.4 (qC, C-113), 162.5 (qC, C-14) and 8.03/162.4 (H-12/C-12) were assigned as an oxalamide and a formamide moiety, respectively, completing the degrees of unsaturation and the structure of **5**. In addition, the mass fragment ion at *m/z* 378 [M − COCONH_2_ + H]^+^ supports the presence of the oxalamide moiety at one side of the molecule. The attachment of these moieties to the terminal amines at C-11 and C-8, respectively, were secured from HMBC cross-peaks of H_2_-11/C-13, H_2_-8/C-12 and H-12/C-8 (Table 2). HSQC and HMBC experiments secured the assignments of protonated and quaternary carbons (Table 2). The chemical shifts of the oxalamide moiety are in good agreement with the reported data in the literature [4]. Thus, compound **5 **was assigned as *N*-(3-(2,6-dibromo-4-(2-formamidoethyl)-phenoxy)propyl)oxalamide. 

Compound **6 **(Figure 1) was purified as an amorphous powder. Its FABMS cluster at *m/z* 450.9/452.9/454.9 required two bromines, and the entire formula C_15_H_21_^79^Br_2_N_2_O_4_ [M + H]^+^ as established from the HRFABMS ion peak at *m/z* 450.9873. The molecular formula of **6** suggests six degrees of unsaturation, being two more than **2**. Comparison of its NMR data (^1^H, ^13^C, HSQC and HMBC) with those of **5** revealed the similarity to the structure of **5** (Table 2). However, instead of the terminal oxalamide moiety at C-11 in **5**, new NMR signals for an ethylcarbamate moiety at δ 159.2 (qC, C-13), 4.05/61.7 (q, CH_2_) (H_2_-14/C-14) and 1.22/15.0 (t, CH_3_) (H_3_-15/C-15) were observed in the spectra of **6**, completing the structure of **6**. The chemical shift values of the signals of the ethylcarbamate moiety on the terminal amine are in good agreement with reported data [2]. Furthermore, the mass fragment ion at *m/z* 378 [M − COOCH_2_CH_3_ + H]^+^ supports the presence of the ethylcarbamate moiety at one side of the molecule. The COSY, HSQC and/or HMBC correlations secured the placement of formamide and ethylcarbamate moieties at C-9 and C-11, respectively. HMBC correlations of H_2_-8/C-12, H-12/C-8, H_2_-11/C-13, H_2_-14/C-13, H_2_-14/C-15, H_3_-15/C-14 (Table 2) supported the assignment of these moieties and their attachment to the terminal amines. Accordingly, compound **6** was assigned as ethyl 3-(2,6-dibromo-4-(2-formamidoethyl)phenoxy)-propylcarbamate. 

Compounds **1** [18], **7** [4], **8** [4], **9** [2], **10** [12], and **11** [12] were identified by comparison of their spectroscopic data with those previously reported in the literature. 

**Table 2:** NMR Spectroscopic Data (Methanol-*d*_4_) for Ceratinines C–E (**4**–**6**).

	Ceratinine C (4)			Ceratinine D (5)			Ceratinine E (6)		
Position	δ_C _(mult.) ^a^	δ_H_ [mult., *J *(Hz)]	HMBC ^b^	δ_C _(mult.) ^a^	δ_H_ [mult., *J* (Hz)]	HMBC ^b^	δ_C _(mult.) ^a^	δ_H_ [mult., *J* (Hz)]	HMBC ^b^
**1**	137.5, C			138.2, C			137.5, C		
**2**	132.9, CH	7.56, s	4, 5, 7	132.9, CH	7.49, s	4, 5, 7	134.2, CH	7.46, s	1, 4, 5, 7
**3**	117.9, C			117.6, C			119.0, C		
**4**	151.5, C			151.4, C			152.9, C		
**5**	117.9, C			117.6, C			119.0, C		
**6**	132.9, CH	7.56, s	4, 5, 7	132.9, CH	7.49, s	4, 5, 7	134.2, CH	7.46, s	1, 4, 5, 7
**7**	31.9, CH	2.91, t (7.0)	1, 6, 8	33.6, CH	2.78, t (7.0)	1, 6, 8	35.0, CH	2.75, t (6.0)	1, 8
**8**	39.6, CH_2_	3.26, t (7.0	1, 7	38.6, CH_2_	3.46, t (7.0)	1, 7, 14	40.0, CH_2_	3.42, t (6.0	1, 7, 12
**9**	70.9, CH_2_	4.09, t (7.0)	4, 10, 11	70.7, CH_2_	4.07, t (7.0)	4, 10, 11	72.0, CH_2_	4.04, t (6.5)	4, 10, 11
**10**	29.1, CH_2_	2.15, quin. (7.0)	9, 11	29.0, CH_2_	2.12, quin. (7.0)	9, 11	31.6, CH_2_	2.01, quin. (6.5)	9, 11
**11**	36.6, CH_2_	3.59, t (7.0)	9, 10, 12	36.6, CH_2_	3.58, t (7.0)	9, 10, 13	39.0, CH_2_	3.36, t (6.5)	9, 10, 13
**12**	160.3, C			162.4, CH	8.03, s	8	163.9, CH	7.99, s	8
**13**				160.4, C			159.2, C		
**14**				162.5, C			61.7, CH_2_	4.05, q (6.5)	13, 15
**15**							15.0, CH_3_	1.22, t (6.5)	14

^a^ Multiplicity of the carbons was assigned from DEPT and HSQC experiment. ^b^ HMBC correlations are from proton(s) stated to the indicated carbon.

### 2.2. Biological Activity of the Compounds

**Figure 4:**
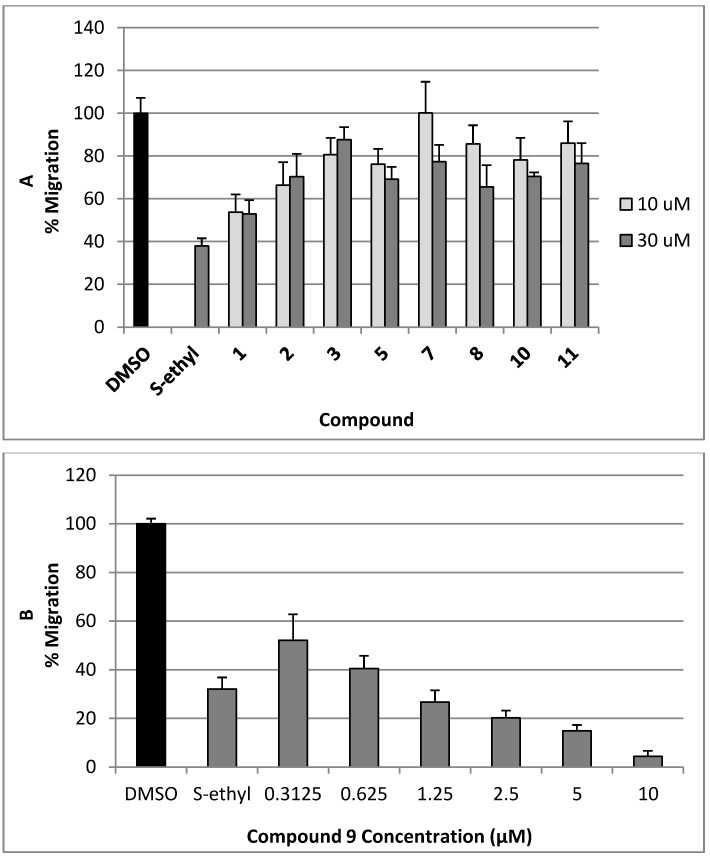
(**A**) The antimigratory activity of the compounds **1**–**3**, **5**, **7**, **8**, **10 **and **11** at two different concentrations in wound healing assay against the highly metastatic MDA-MB-231 human breast cancer cell line; (**B**) Dose response activity of compound **9** in WHA. Each concentration was run in triplicate and expressed as the mean ± SEM.

**Figure 5:**
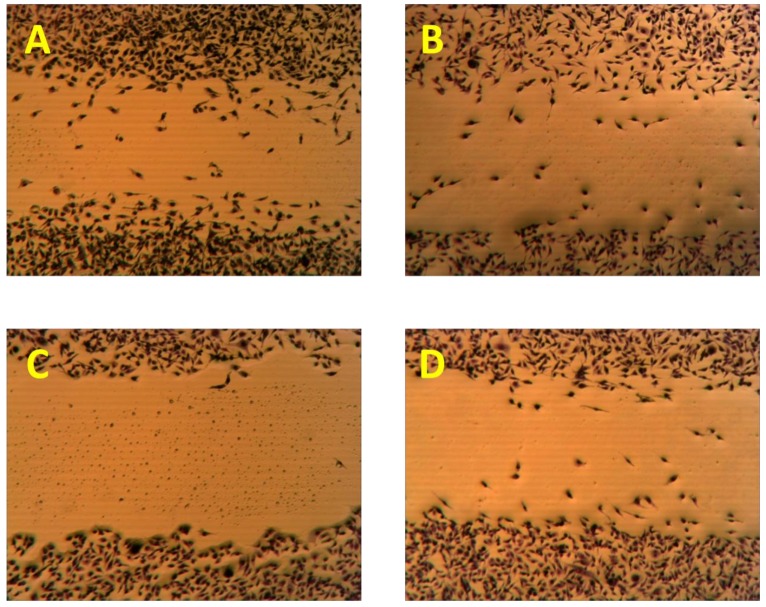
The antimigratory activity of the subereamolline A (**9**) using a wound-healing assay at two different concentrations 5 µM (**C**), and 0.31 µM (**D**) as compared to vehicle control (**A**) and positive control (**B**).

**Figure 6:**
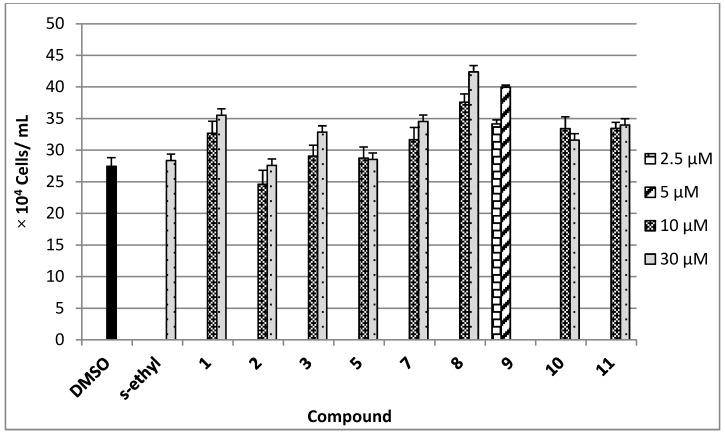
Viability of the highly metastatic MDA-MB-231 human breast cancer cells in the presence of 10 µM and 30 µM doses of **1**–**3**, **7**, **8**, **10**, **11 **and 2.5 µM and 5 µM of **9 **after 24 h incubation in serum free media containing 0.5% fetal bovine serum. Each concentration was run in triplicate and the data are expressed as the mean ± SEM. 4-*S*-Ethylphenylmethylene hydantoin (*S*-ethyl) was used as a positive control [22,23].

The antimigratory activity of compounds **1**–**3**, **5**, and **7**–**11 **against the highly metastatic MDA-MB-231 human breast cancer cell line was evaluated using the wound-healing assay (WHA, Figure 4) [21,22]. The wound-healing assay is a simple tool to investigate the *in vitro* directional cell migration [21,22,23]. The scratched tumor cell monolayer heals the wound in a specific fashion. The ability of the compounds to inhibit the migration of the highly metastatic MDA-MB-231 human breast cancer cells into the wound is measured by counting the number of the cells in the wound after 24 h incubation. The higher the antimigratory activity of the compound, the lower the number of the cells migrated into the wound. Therefore, this assay is widely used to study cell migration. However, little number of the cells may be found in the wound due to the effect of the compounds on their viability. The antimigratory activity of all compounds was assessed in the wound-healing assay at a 10 and 30 µM doses (Figure 4A), except compound **9**, which was tested at several concentrations below 10 µM (Figure 4B and Figure 5) against MDA-MB-231 cells**, **to avoid false positive activity due to their possible cytotoxicity. The activity was compared to a 30 µM dose of the antimetastatic lead 4-*S*-ethylphenylmethylene hydantoin (*S*-ethyl), which was discovered based on marine natural products [22,23]. The effect of the compounds **1**–**3**, **5**, and **7**–**11 **on the viability of the highly metastatic MDA-MB-231 human breast cancer cells was evaluated by MTT assay (Figure 6) [21,22]. The compounds showed no effect on the viability of MDA-MB-231 cells up to a 50 µM dose, indicating the lack of cytotoxicity toward this cell line. Only compounds **9** showed cytotoxic activity against MDA-MB-231 cells at 30 µM but showed no toxicity at 10 µM.

Initially, subereamolline A (**9**) showed significant antimigratory activity at 10 µM (Figure 4B). Therefore, it was reevaluated at six different concentrations (10, 5, 2.5, 1.25, 0.625, and 0.3125 µM) in order to calculate its IC_50_ value (Figure 5). At the nanomolar dose level (0.3125 µM), the percent migration of the highly metastatic MDA-MB-231 human breast cancer cell line was about 52%. Therefore, it is expected its IC_50_ value will be around 400 nM. Aerothionin (**10**) with terminal 1-oxa-2-azaspiro[4.5]deca-2,6,8-trienecarboxamido groups at each side showed no activity. This clearly indicates the importance of the terminal ethyl carbamate moiety in subereamolline A because it is identical in structure to **10** except this moiety. This may also imply the need for low molecular size group as bulky groups like 1-oxa-2-azaspiro[4.5]deca-2,6,8-trienecarboxamido may hinder the binding at the target receptor. Homoaerothionin (**11**) was also inactive; suggesting the 4-carbons *n*-butyl group connecting the two NH groups was preferred over the 5-carbon *n*-pentyl group. 

Moloka’iamine (3-(4-(2-aminoethyl)-2,6-dibromophenoxy)propan-1-amine, **1**) was the most active, inhibiting >50% of the migration at 30 and 10 μM, while ceratinine C ((2*R*,3*S*)-3-(1-(5,7-dibromo-4-methylindolin-6-yloxy)ethyl)oxiran-2-amine, **3**) was the least active (Figure 4). This suggests the preference of the dibromophenoxy group over the dibromo-4-methylindolin-6-yloxy group for the antimigratory activity. The low activity of hydroxymoloka’iamine (**7**) compared to **1** suggested that C-8 hydroxy group adversely affect the activity.

**Figure 7:**
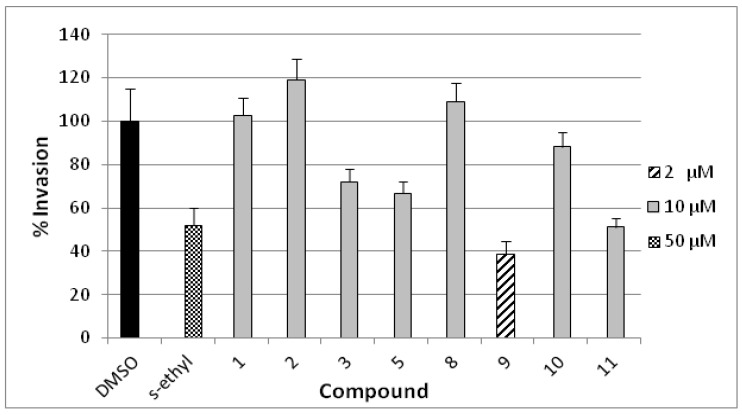
Anti-invasive activities of 10 μM dose of **1**–**3**, **5**, **8**, **10**, **11** and 2 μM of **9** in the Cultrex^®^ BME cell invasion assay against the highly metastatic MDA-MB-231 human breast cancer cell line. 4-*S*-Ethylphenylmethylene hydantoin (*S*-ethyl) was used as a positive control (50 µM) [22,23]. Each concentration was run in triplicate and the data are expressed as the mean ± SEM.

The anti-invasive activity of compounds **1**–**3**, **5**, and **8**–**11** was assessed using the Cultrex^®^ BME cell invasion assay against the highly metastatic MDA-MB-231 human breast cancer cell line (Figure 7) [24]. The activity was compared to a 50 µM dose of the antimetastatic lead 4-*S*-ethylphenylmethylene hydantoin (*S*-ethyl) [22,23]. The compounds have been screened at 10 µM dose, homoaerothionin (**11**) has shown 50% invasion whereas compounds **3** and **5** showed medium activity. Compound **9** was cytotoxic at the tested concentration and, thus, it was re-evaluated at 2 µM dose which has shown 38% invasion. Subereamolline A (**9**) was screened at four different concentrations (2, 1.5, 1, and 0.5 µM) and its IC_50_ value was 1.7 µM. This clearly shows the potential of subereamolline A as possible scaffold for the future design of breast cancer migration and invasion inhibitors. 

## 3. Experimental Section

### 3.1. General Experimental Procedures

Optical rotations were recorded on a JASCO DIP-730 digital polarimeter. Ultraviolet spectra were recorded on a Hitachi 300 spectrometer. NMR spectra were obtained in CD_3_OD on Bruker Avance DRX 400 Spectrometers at 400 MHz for ^1^H NMR and 100 MHz for ^13^C NMR. NMR chemical shifts are expressed in parts per million (ppm) referenced to CD_3_OD solvent signals (δ 3.29 for ^1^H and δ 49.0 for ^13^C) or DMSO-d_6_ signals (2.49 ppm for ^1^H and 39.9 ppm for ^13^C). Positive ion FAB mass spectral data were obtained with a Micromass Q-tof equipped with lockspray mass spectrometer using Leucine Enkaphalin at *m/z* 556.2771 [M + H]^+^ as a reference mass. Pre-coated silica gel G-25 UV_254_ plates were used for thin layer chromatography and silica gel 60 Å, 230–400 μm mesh (E. Merck) and Sephadex LH-20 (Pharmacia) employed for column chromatography.

### 3.2. Animal Materials

#### 3.2.1. *Pseudoceratina arabica*

The marine sponge *P. arabica* was collected by hands using SCUBA from Hurghada at the Egyptian Red Sea coast at depths between 17 and 25 m in 2011. The sponge is massively encrusting with a conulose surface. The color of the living sponge underwater is yellow-green with a bright yellow interior. The preserved sample changed to a blackish-green color, and the alcohol became discolored to dark green as well. The surface conules are bluntly rounded, 2–5 mm apart with the consistency firmly compressible and rubbery. The voucher fragment is 10.0 × 4.0 × 1.0 cm. The skeleton consists of sparse irregular fibers consisting only of pith. The outline and branching is irregular, and thickness varies between 80 and 300 μm. The specimen conforms to the description of the type from the Eritrean Red Sea The identity of the sponges were confirmed by Dr. R. van Soest and vouchers were kept in the collections of the Zoological Museum of the University of Amsterdam under registration number 17951. Other voucher specimen was deposited in the Red Sea Invertebrates Collection of the Department of Pharmacognosy, Suez Canal University under the code number DY-2011-61.

#### 3.2.2. *Suberea mollis*

The sponge *Suberea mollis* was collected from Hurghada at depths between 15 and 25 m in 2011. The sponge is cylindrical in shape with conulose surface. The conules were low but sharp due to projecting strong fibers, about 8–10 mm apart. The oscules are large, approximately 1.0 cm in diameter, positioned at the summit of the fragment. In life, the sponge is green in color with a yellowish interior. In a preserved condition it turned black. The interior of the sponge is cavernous. The ectosomal region is a distinctly denser mass of collagen and crowded large spherulous cells, whereas deeper in the body the organic parts are only lightly collagenous and they are charged with many small calcareous nodules. The skeleton consists of thick pitched fibers, which run for long distances without branching or anastomosing. The fibers measure approximately 400 μm in diameter, of which the pith occupies 75%. The bark consists of several thick laminae of amber colored sponging, This sponge conforms in most aspects (shape, surface characters and fibers) to the description of the type of *Suberea mollis *(Row), 1911 (as *Aplysina*) (class Demospongiae, order Verongida, family Aplysinellidae). The identity of the sponges was confirmed by Dr. R. van Soest and a voucher was kept in the collections of the Zoological Museum of the University of Amsterdam under registration number 16621. Other voucher specimen was deposited in the Red Sea Invertebrates Collection of the Department of Pharmacognosy, Suez Canal University under the code number DY-2011-8. 

### 3.3. Extraction and Isolation

#### 3.3.1. Isolation of Compounds **1**–**8**

Freshly collected specimens of the sponge *P. arabica* (1.65 kg) were frozen immediately after collection on site. The frozen sponge materials were crushed and then extracted with MeOH (3 × 3000 mL) at room temperature. The combined crude extracts were evaporated under reduced pressure to give a brown viscous crude extract (7.9 g). The extract was partitioned between 90% MeOH and *n*-hexane and the mother liquor was diluted with H_2_O to 60% MeOH and was successively extracted with CH_2_Cl_2_, EtOAc and *n*-butanol. The polar *n*-butanol fraction, (1.8 g) which was rich in salt, was dissolved in MeOH, filtered and the MeOH-soluble fraction (750 mg) was fractionated on ODS flash column using H_2_O/MeOH gradient to afford three promising fractions (52, 65 and 63 mg). The fractions were further purified separately several times on C-18 Sep-Pak Vac cartridge (Waters, 10 g) using H_2_O/MeOH gradient. Final purification of the sub-fractions was achieved by HPLC using C18-AR II (10 × 250 mm) column using 5% MeOH in H_2_O to give compounds **1** (15 mg), **2** (3.5 mg), **3** (7.5 mg) and **7** (6.5 mg). The less polar CH_2_Cl_2_ extract (3.8 g) was dissolved in dilute HCl. The acidic solution was extracted several times with CH_2_Cl_2_ to give 2.6 g of brown residue. This extract was subjected to VLC using normal phase silica gel and *n*-hexane/CH_2_Cl_2_/MeOH gradient to afford six main fractions. Fraction 2 (266 mg) was chromatographed on normal phase silica gel using *n*-hexane/CHCl_3_/MeOH to give four subtractions, F2A (48.9 mg), F2B (60 mg), F2C (54.8 mg) and F2D (36.2 mg). These fractions were subjected, separately, to final HPLC purification on reversed phase HPLC using semipreparative C18-AR II (10 × 250 mm) column using 75% MeOH in H_2_O. Fraction F2A gave **4** (4.2 mg) and **8** (3.2 mg), while **6** (7.2 mg) was purified from fraction F2C. Fraction 3 (340 mg) was chromatographed on normal phase silica gel using *n*-hexane/CHCl_3_/methanol to afford two subtractions, F3A (96 mg) and F3B (87 mg). Fraction F3A was subjected to size exclusion chromatography on Sephadex LH-20 using MeOH followed by HPLC purification on C18-AR II (10 × 250 mm) using 20% MeOH in H_2_O to yield **5** (5.5 mg).

#### 3.3.2. Isolation of Compounds **9**–**11**

Freshly collected specimens of the sponge *S. mollis* (540 g) were chopped into small pieces and extracted with MeOH. The extract was concentrated and defatted with *n*-hexane. The mother liquor was concentrated to yield a viscous brown residue (2.8 g). The residue was applied to a silica gel column, eluted with hexanes/CH_2_Cl_2_/MeOH gradient to yield three fractions. The active fraction (540 mg) was subjected to a Sephadex LH-20 column eluted with MeOH-CH_2_Cl_2_ (1:1) to produce three subfractions. The second active fraction (88 mg) was purified on RP C18 semi-preparative HPLC column using 55% MeCN in H_2_O to afford compounds **9** (3.5 mg), **10** (4.5 mg) and **11** (3.8 mg).

Ceratinine A (**2**): Yellow oil; α^25^_D_= −22 (*c* 0.47, MeOH); UV (MeOH) λ_max_ nm (log ε) 275 (2.18), 224 (3.67), 217 (3.66); ^1^H and ^13^C NMR data, see Table 1; positive HRFABMS *m/z *380.9822 (calculated for C_12_H_19_^79^Br_2_N_2_O_2_, [M + H]^+^, 380.9813).

Ceratinine B (**3**): Off-white amorphous powder; α^25^_D_ = −16 (*c* 0.25, MeOH); UV (MeOH) λ_max_ nm (log ε) 337 (3.40), 299 (3.71); ^1^H and ^13^C NMR data, see Table 1; positive HRFABMS *m/z *390.9683 (calculated for C_13_H_17_^79^Br_2_N_2_O_2_, [M + H]^+^, 390.9657).

Ceratinine C (**4**): Off-white amorphous powder; UV (MeOH) λ_max_ nm (log ε) 282 (2.27), 276 (2.34), 207 (3.71); ^1^H and ^13^C NMR data, see Table 2; positive HRFABMS *m/z *393.9772 (calculated for C_12_H_18_^79^Br_2_N_3_O_2_, [M + H]^+^, 393.9772).

Ceratinine D (**5**): Off-white amorphous powder; UV (MeOH) λ_max_ nm (log ε) 281 (2.25), 275 (2.35), 209 (3.70); ^1^H and ^13^C NMR data, see Table 2; positive HRFABMS *m/z *449.9655 (calculated for C_14_H_18_^79^Br_2_N_3_O_4_, [M + H]^+^, 449.9664).

Ceratinine E (**6**): Amorphous white powder; UV (MeOH) λ_max_ nm (log ε) 276 (2.35), 208 (3.70); ^1^H and ^13^C NMR data, see Table 2; positive HRFABMS *m/z *450.9873 (calculated for C_15_H_21_^79^Br_2_N_2_O_4_, [M + H]^+^, 450.9868).

## 4. Bioassay Methods

### 4.1. Wound-Healing Assay

The highly metastatic human MDA-MB-231 breast cancer cells were cultured in RPMI 1640 medium containing 10 mM HEPES, 4 mM L-glutamine, 10% fetal bovine serum, penicillin (100 IU/mL), and streptomycin (50 μg/mL), and grown in a 5% CO_2_ atmosphere at 37 °C [21]. Cells were plated onto sterile 24-well and allowed to recover for a confluent cell monolayer formed in each well (>95% confluence). Wounds were then inflicted to each cell monolayer using a sterile 200 μL pipette tip. Media were removed, cells were washed twice with PBS, and then fresh media containing test compounds were added to each well. Test compounds were prepared in DMSO stock solution (50 mM). The required test compound concentrations were prepared in serum free media containing 0.5% fetal bovine serum. Initially, the ability of the compounds to inhibit the migration of the cells into the wound was performed at 10 and 30 µM concentrations. The compounds that show promising migration inhibitory activity were tested at 6 non-toxic concentrations, prepared by serial dilution, each in triplicate using DMSO as negative control. The incubation was carried out for 24 h, after which media was removed and cells were fixed and stained using Diff Quick staining (Dade Behring Diagnostics, Aguada, Puerto Rico) [22,23]. The number of cells migrated on the scratched wound were counted under the microscope in three or more randomly selected fields (magnification: 40×). Final results are expressed as mean per 40× field.

### 4.2. Cytotoxicity Assay

The highly metastatic MDA-MB-231 human breast cancer cells were cultured in RPMI 1640 medium containing 10 mM HEPES, 4 mM L-glutamine, 10% fetal bovine serum, penicillin (100 IU/mL), and streptomycin (50 μg/mL), and incubated in a 5% CO_2_ atmosphere at 37 °C. For subculturing, cells were rinsed twice with sterile Ca^2+^ and Mg^2+^-free phosphate buffered saline (PBS) and incubated in 0.25% trypsin containing 0.025% EDTA in PBS for 5 min at 37 °C. The released cells were centrifuged, resuspended in fresh media and counted using hemocytometer. Cells were plated onto sterile 96-well plates, at an initial cell count of 20 × 10^4^ cells/mL and allowed for complete attachment overnight. A stock solution of the compounds was prepared in DMSO. About 2 µL of each stock solution was transferred to 998 µL of serum-free medium containing 0.5% fetal bovine serum to obtain 100 µM concentrations (0.2% DMSO). Serial dilutions were then conducted to obtain the desired concentrations for the assay. Cell viable number was determined by the 3-(4,5-dimethylthiazol-2yl)-2,5-diphenyl tetrazolium bromide (MTT) colorimetric assay after incubation period of 24 h. On the assay day, treatment medium was replaced with fresh control medium before the addition of 1 mg/mL MTT (50 µL/well), and the cells in 96-well plates were incubated at 37 °C for 4 h. The medium was removed, and the MTT crystals were dissolved in DMSO (100 µL/well). The optical density of each sample was read at 570 nm on a microplate reader (Synergy™ 2, Biotek Instruments, Inc.), against a blank prepared from cell-free cultures. The number of cells/well was calculated against a standard curve prepared by plating various concentrations of cells, as determined by hemocytometer, at the start of each experiment. 

### 4.3. Cultrex^®^ BME Cell Invasion Assay

Anti-invasive activities were measured using Cultrex^®^ BME cell invasion assay [24]. About 50 μL of basement membrane extract (BME) coat was added per well of the top chamber. After an overnight incubation at 37 °C in a 5% CO_2_ atmosphere, 50,000/50 μL of MDA-MB-231 cells in 0.5% FBS RPMI medium were added per well of the top chamber. 150 μL of RPMI medium was then added to the lower chamber. Media contained 10% FBS and penicillin/streptomycin as well as fibronectin (1 μL/mL) and *N*-formyl-Met-Leu-Phe (10 nM) as chemoattractants. Test compounds were prepared at 6× the desired concentration and 10 μL of each of the compounds was added per well of the top chamber. Cells were incubated at 37 °C under 5% CO_2_ which allowed for cell migration from the top to the lower chamber. After 24 h, the top and bottom chambers were aspirated and washed with washing buffer supplied within the kit. About 100 μL of cell dissociation solution/calcein-AM solution was added to the bottom chamber and incubated at 37 °C under 5% CO_2_ for 1 h. The cells internalize calcein-AM, and the intracellular esterases cleave the acetomethylester (AM) moiety to generate free calcein. Fluorescence of the samples was determined at λ_excitation_ 485 nm and λ_emission_ 528 nm using an ELISA plate reader (BioTek, VT, USA). The numbers of cells that invaded through the BME coat were calculated using a standard curve. 

## 5. Conclusions

In conclusion, the investigation of a new collection of the Red Sea Verongid sponge *Pseudoceratina arabica* afforded five new alkaloids: ceratinines A–E (**2**–**6**) together with moloka’iamine (**1**), hydroxymoloka’iamine (**7**) and moloka’iakitamide (**8**). In addition, the antimigratory active fraction of the Verongid sponge *Suberea mollis* afforded subereamolline A (**9**), aerothionin (**10**) and homoaerothionin (**11**). The antimigratory activity of compounds **1**–**3**, **5**, and **7**–**11 **against the highly metastatic MDA-MB-231 human breast cancer cell line was then evaluated using the wound-healing assay. Moreover, the anti-invasive activity of compounds **1**–**3**, **5**, and **8**–**11** was assessed using the Cultrex^®^ BME cell invasion assay against the highly metastatic MDA-MB-231 human breast cancer cell line. Subereamolline A (**9**) potently inhibited the migration and invasion of the highly metastatic human breast cancer cells MDA-MB-231 at the nanomolar doses. These results display the potential of subereamolline A and related brominated alkaloids as a possible scaffold for the future design of breast cancer migration and invasion inhibitors. 

## Supplementary Files

Supplementary File 1: PDF-Document (PDF, 438 KB)
