# Supplementary material for: Subereamolline A as a Potent Breast Cancer Migration, Invasion and Proliferation Inhibitor and Bioactive Dibrominated Alkaloids from the Red Sea Sponge Pseudoceratina arabica
Source: Mar Drugs. 2012 Nov 8;10(11):2492–508. doi: 10.3390/md10112492 (PMC3509531; doi:10.3390/md10112492)

Supplementary Information

**Figure S1.**	^{1}H NMR spectrum of compound **1** (CD_3OD).	2
**Figure S2.**	^{13}C NMR spectrum of compound **1** (CD_3OD).	2
**Figure S3.**	^{1}H NMR spectrum of compound **2** (CD_3OD).	3
**Figure S4.**	^{13}C NMR spectrum of compound **2** (CD_3OD).	3
**Figure S5.**	^{1}H NMR spectrum of compound **3** (CD_3OD).	4
**Figure S6.**	^{1}H NMR spectrum of compound **3** (DMSO-d_6).	4
**Figure S7.**	^{13}C NMR spectrum of compound **3** (CD_3OD).	4
**Figure S8.**	COSY spectrum of compound **3** (CD_3OD).	5
**Figure S9.**	HSQC spectrum of compound **3** (CD_3OD).	5
**Figure S10.**	HMBC Spectrum of compound **3** (CD_3OD).	6
**Figure S11.**	NOESY Spectrum of compound **3** (CD_3OD).	6
**Figure S12.**	^{1}H NMR spectrum of compound **4** (CD_3OD).	7
**Figure S13.**	^{13}C NMR spectrum of compound **4** (CD_3OD).	7
**Figure S14.**	^{1}H NMR spectrum of compound **5** (CD_3OD).	8
**Figure S15.**	^{13}C NMR spectrum of compound **5** (CD_3OD).	8
**Figure S16.**	^{1}H NMR spectrum of compound **6** (CD_3OD).	9
**Figure S17.**	^{13}C NMR spectrum of compound **6** (CD_3OD).	9

Figure S1. ^{1}H NMR spectrum of compound **1** (CD_3OD).

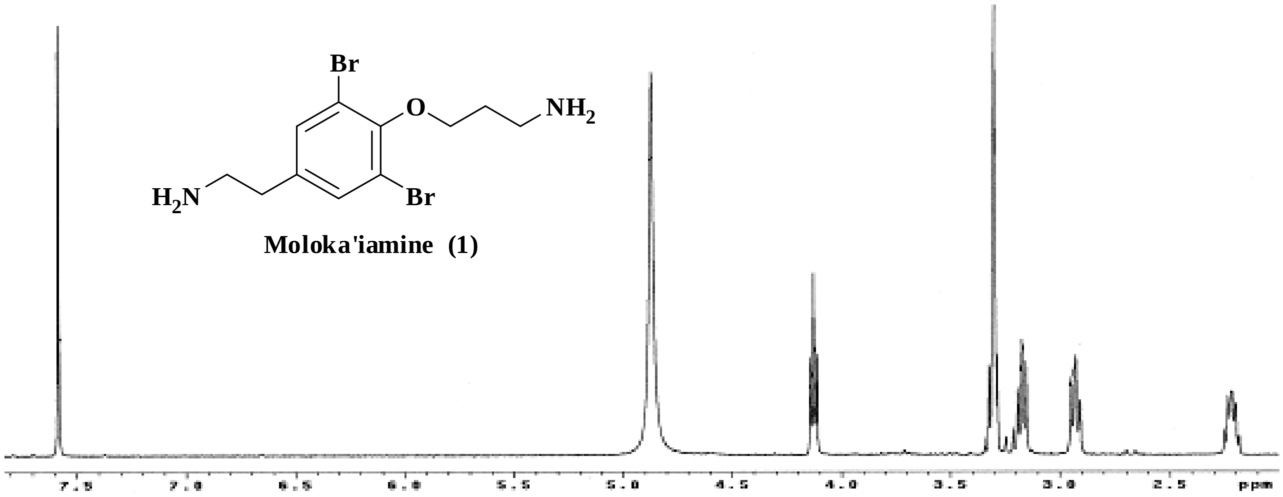

Figure S2. ^{13}C NMR spectrum of compound **1** (CD_3OD).

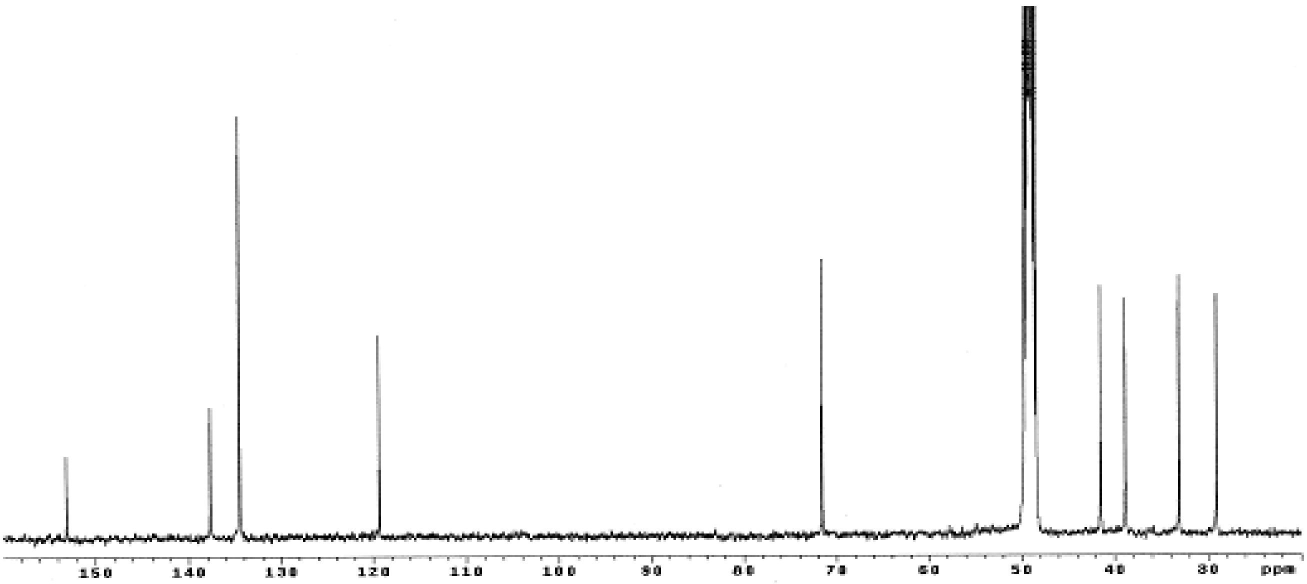

Figure S3. ^{1}H NMR spectrum of compound **2** (CD_3OD).

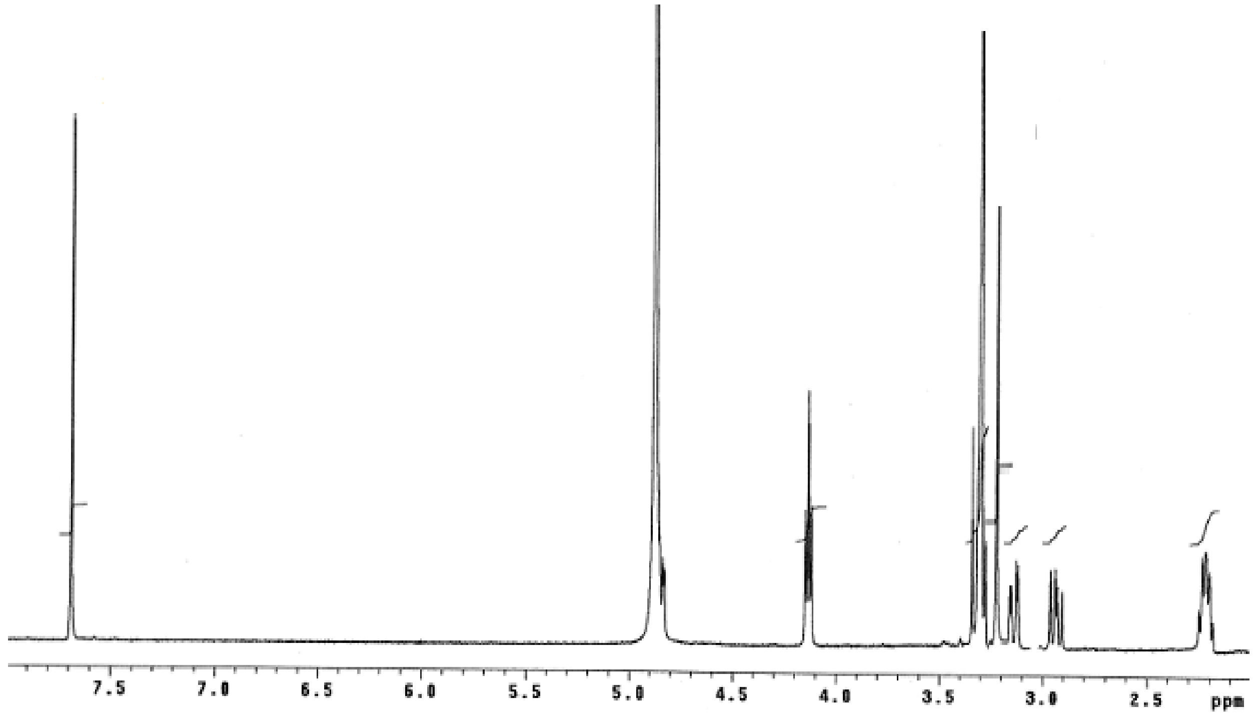

Figure S4. ^{13}C NMR spectrum of compound **2** (CD_3OD).

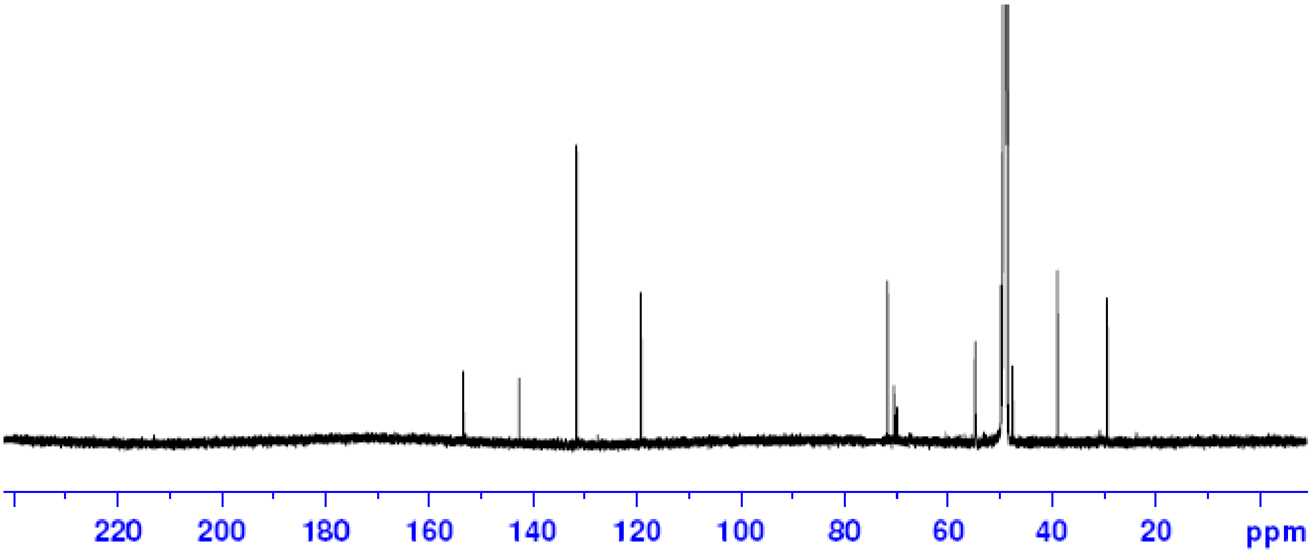

Figure S5. ^{1}H NMR spectrum of compound **3** (CD_3OD).

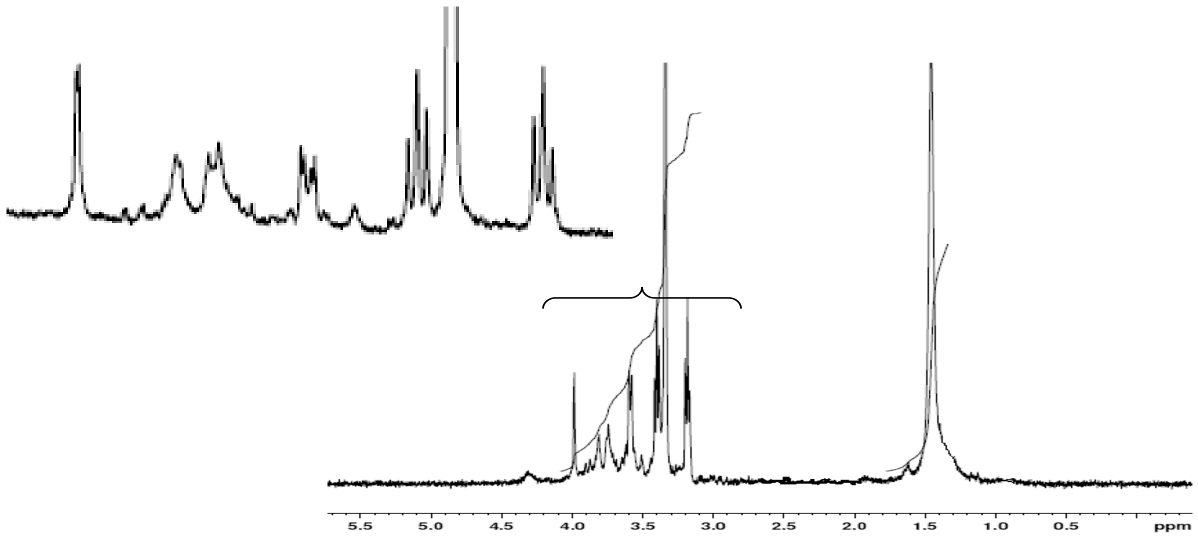

Figure S6. ^{1}H NMR spectrum of compound **3** (DMSO-d_6).

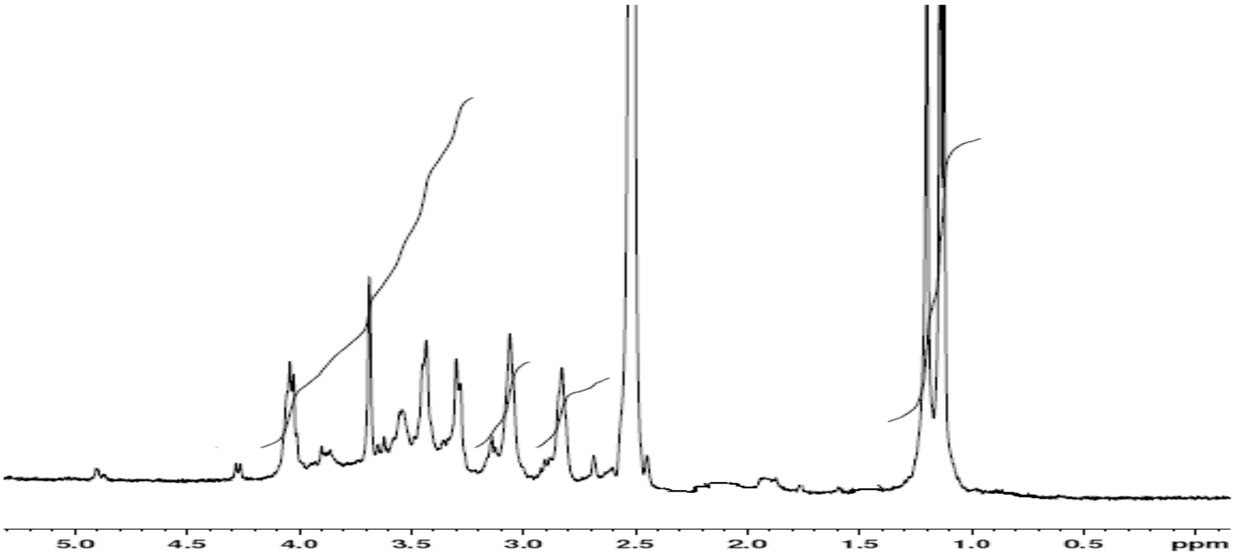

Figure S7. ^{13}C NMR spectrum of compound **3** (CD_3OD).

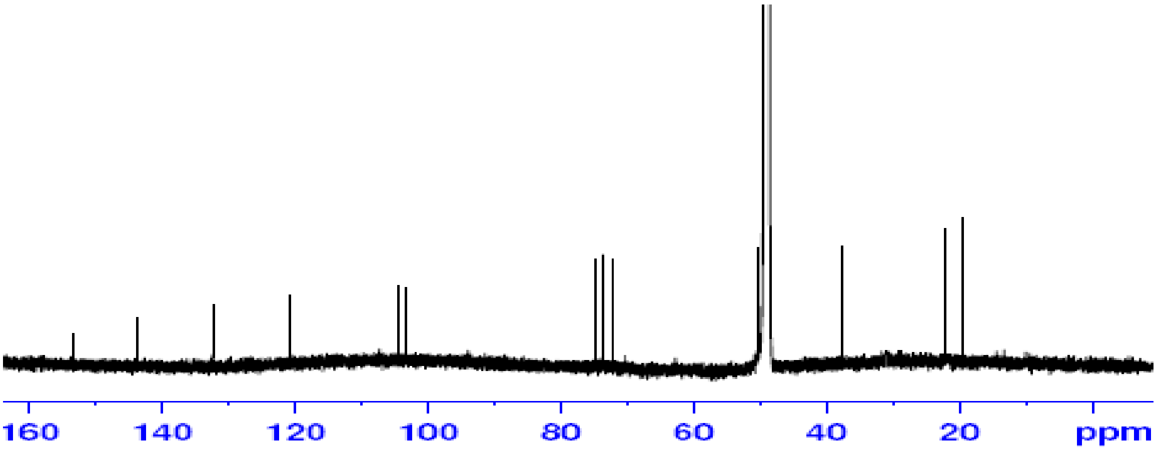

Figure S8. COSY spectrum of compound **3** (CD_3OD).

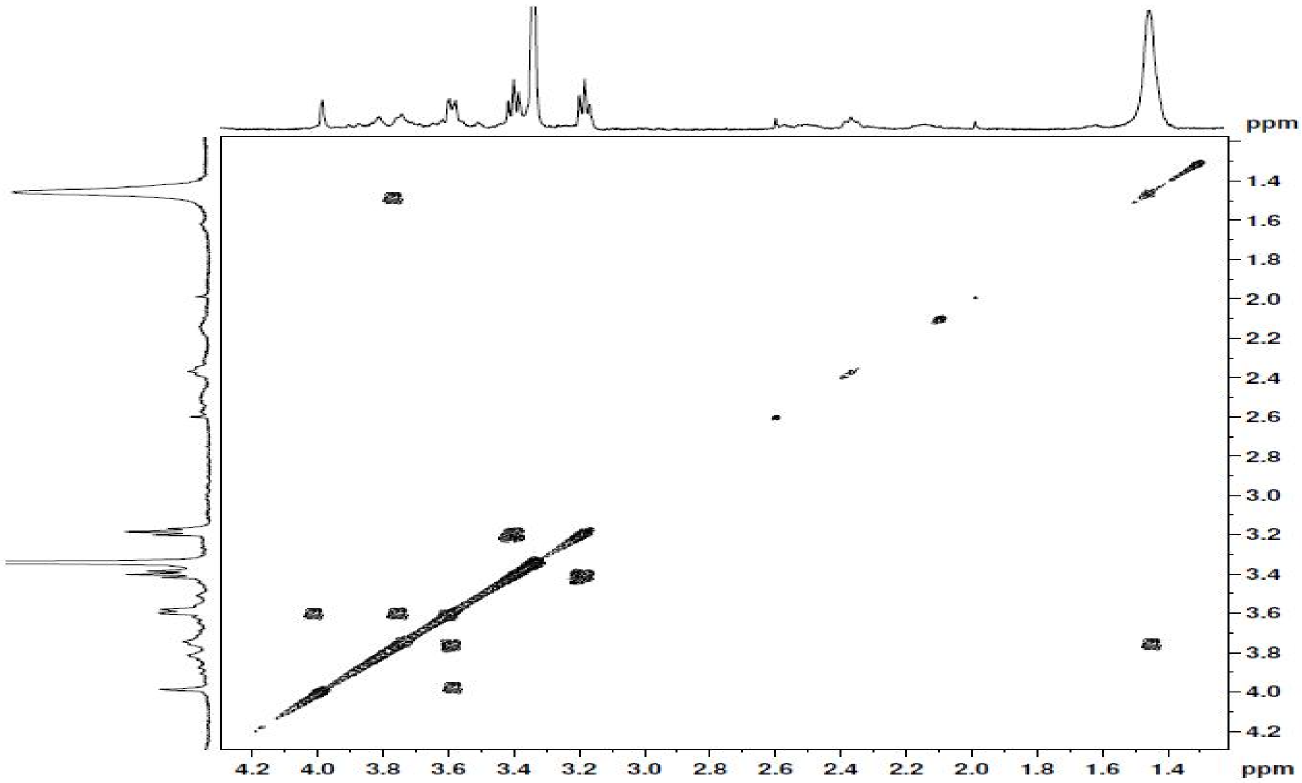

Figure S9. HSQC spectrum of compound **3** (CD_3OD).

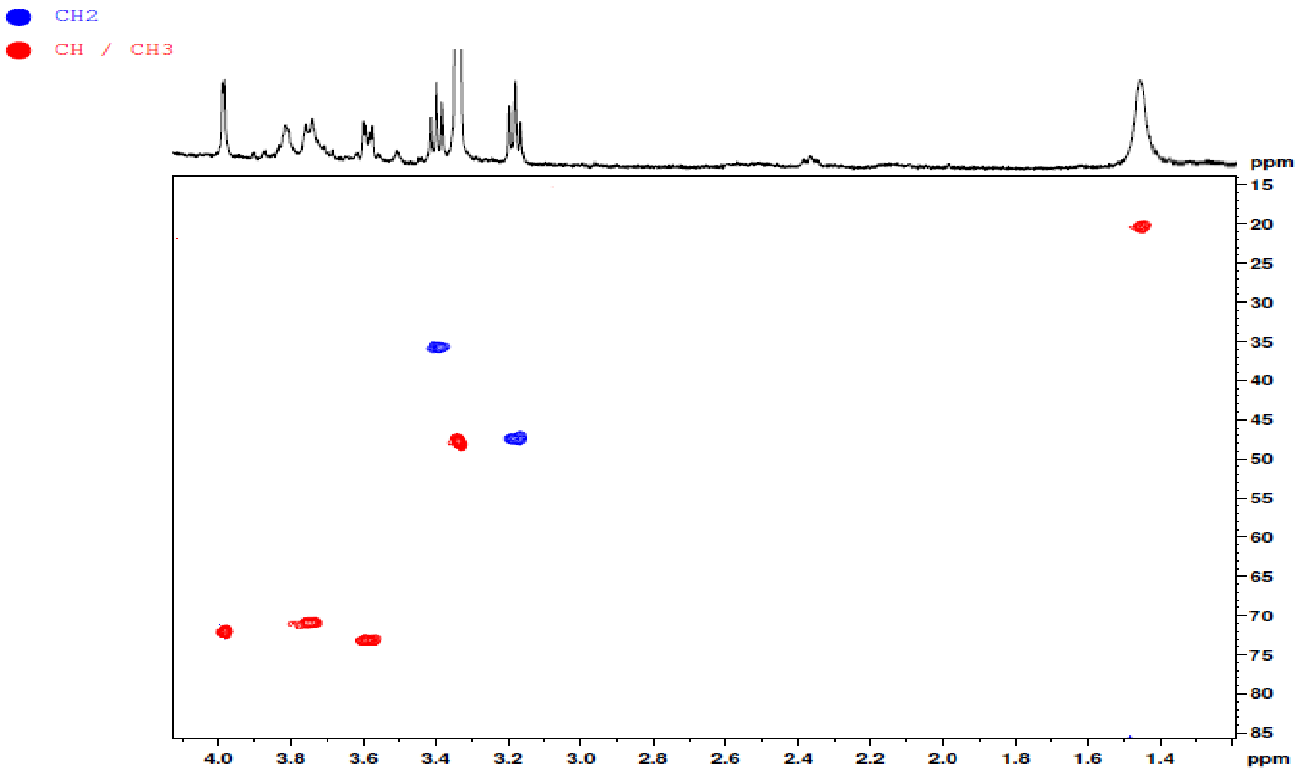

Figure S10. HMBC Spectrum of compound **3** (CD_3OD).

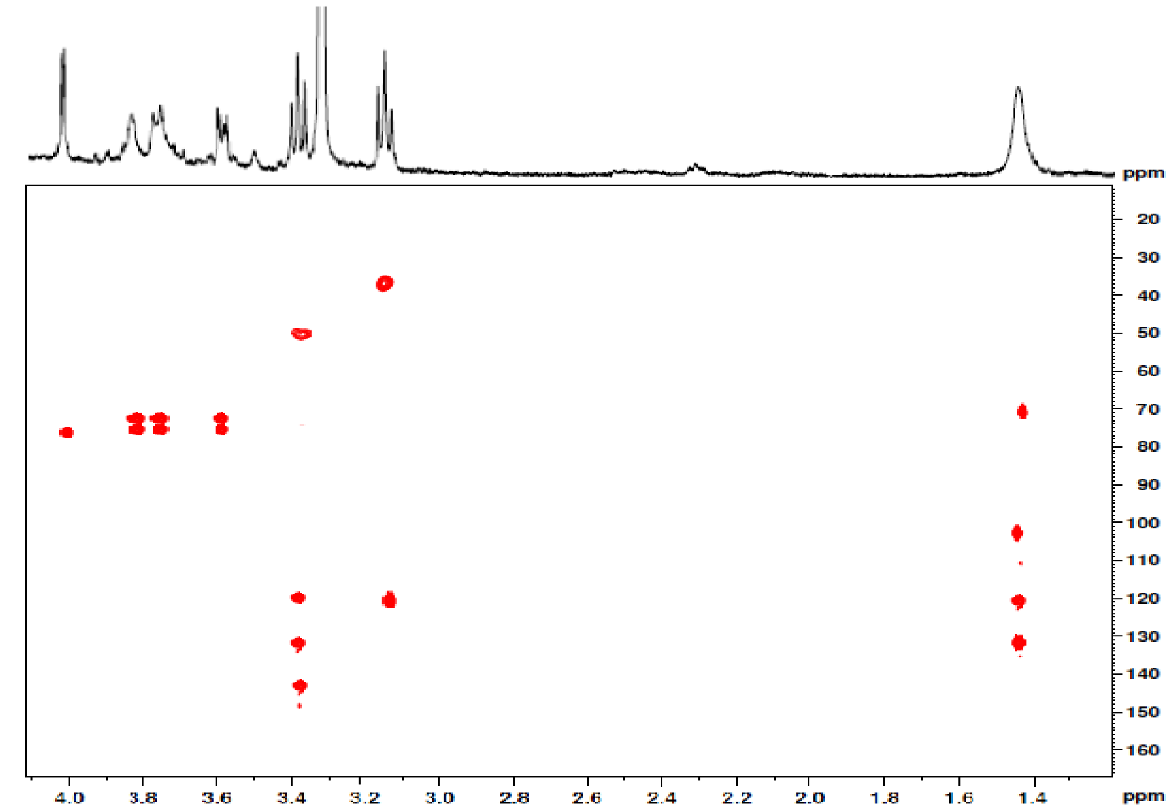

Figure S11. NOESY Spectrum of compound **3** (CD_3OD).

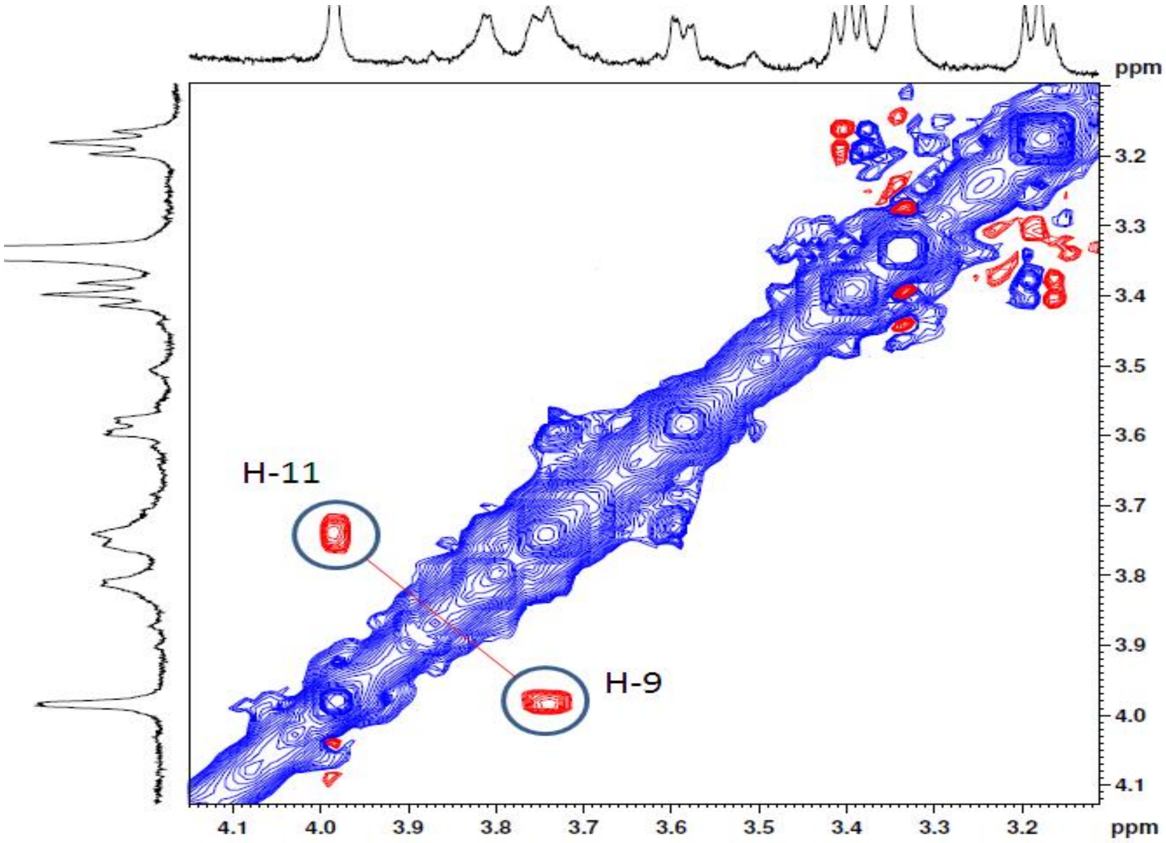

Figure S12. ^{1}H NMR spectrum of compound **4** (CD_3OD).

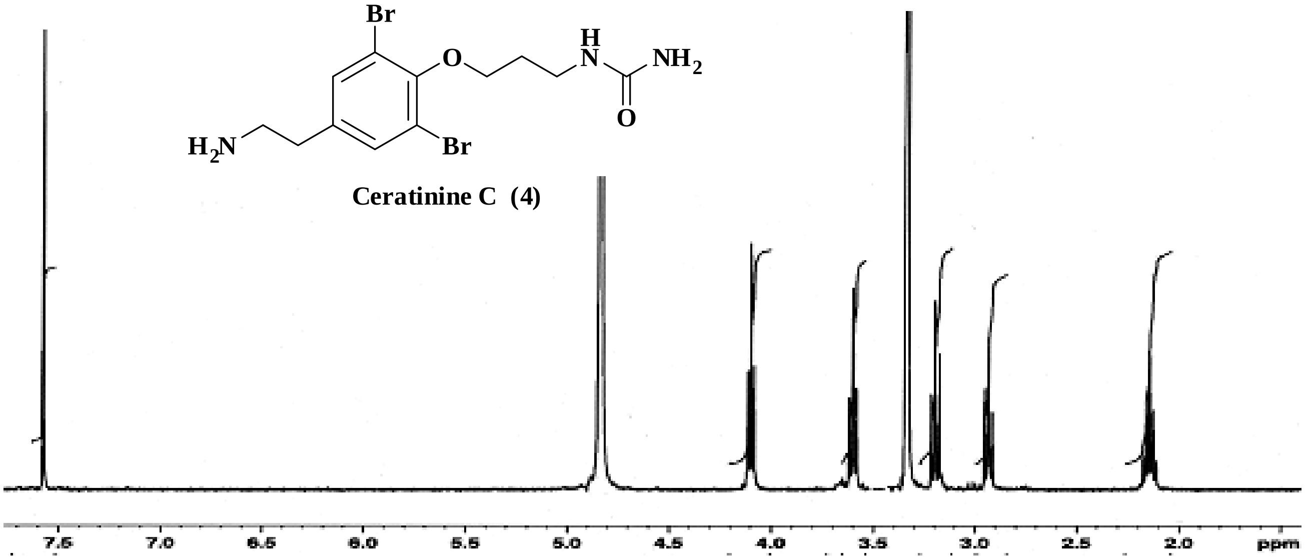

Figure S13. ^{13}C NMR spectrum of compound **4** (CD_3OD).

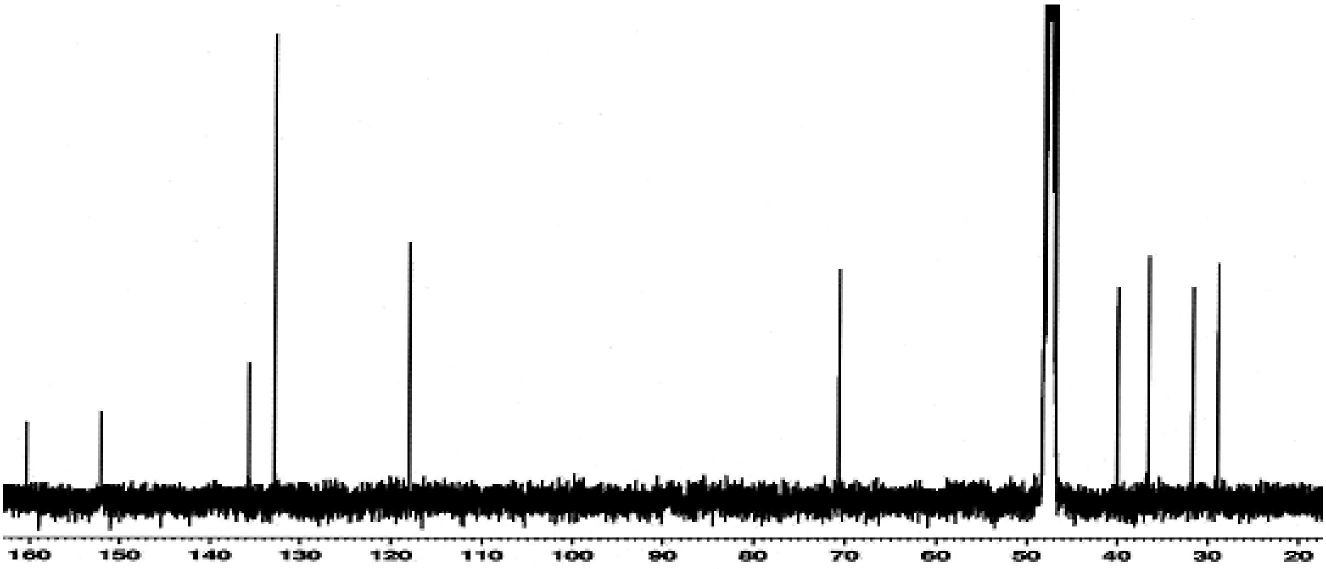

Figure S14. ^{1}H NMR spectrum of compound **5** (CD_3OD).

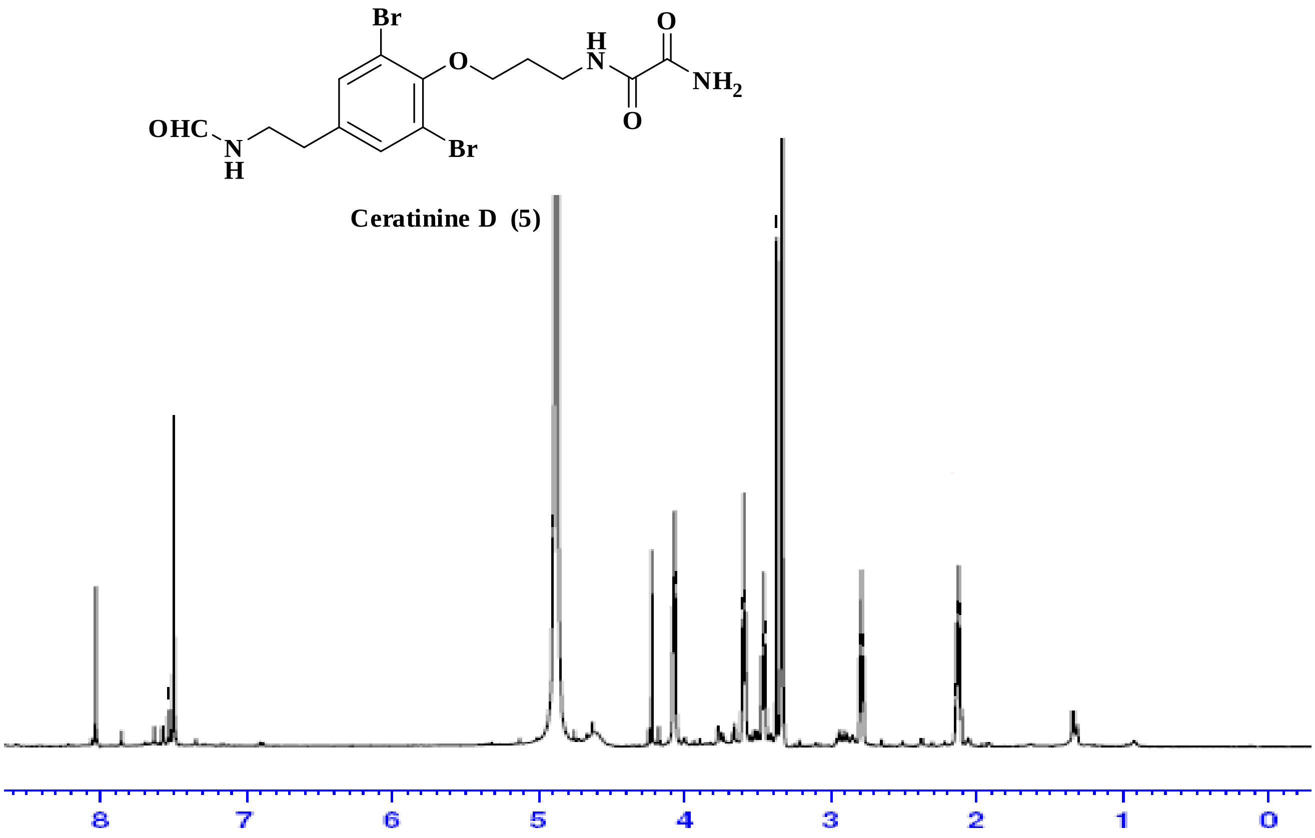

Figure S15. ^{13}C NMR spectrum of compound **5** (CD_3OD).

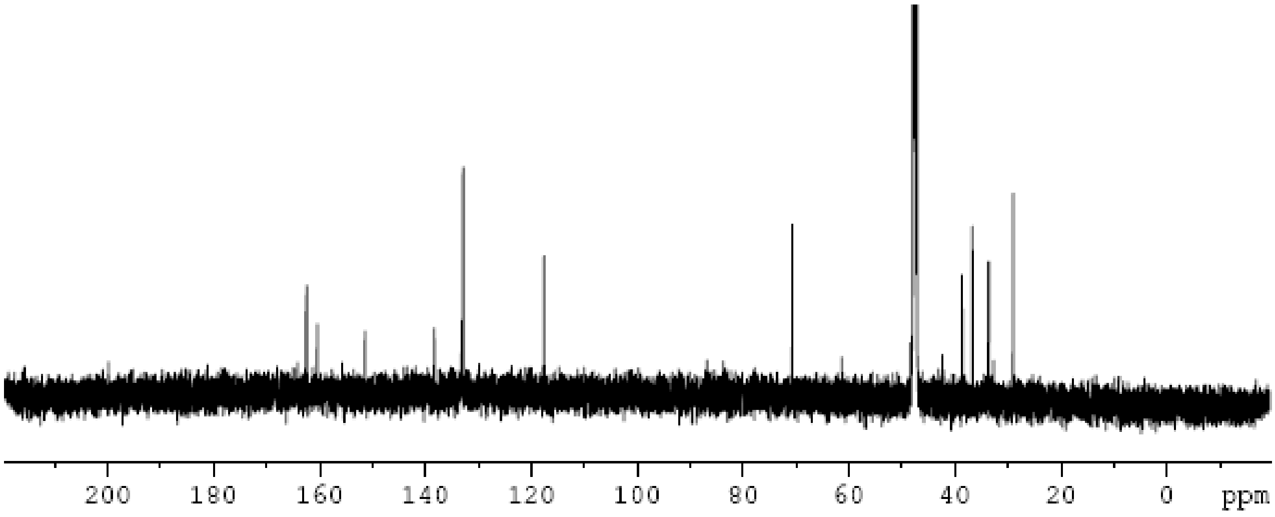

Figure S16. ^{1}H NMR spectrum of compound **6** (CD_3OD).

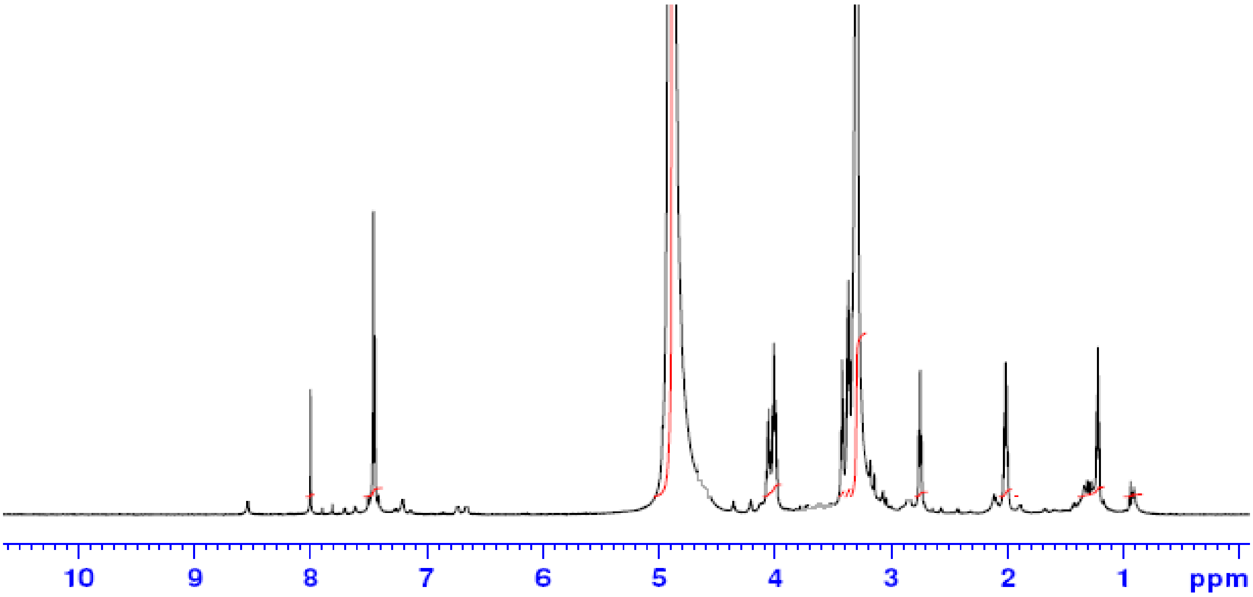

Figure S17. ^{13}C NMR spectrum of compound **6** (CD_3OD).

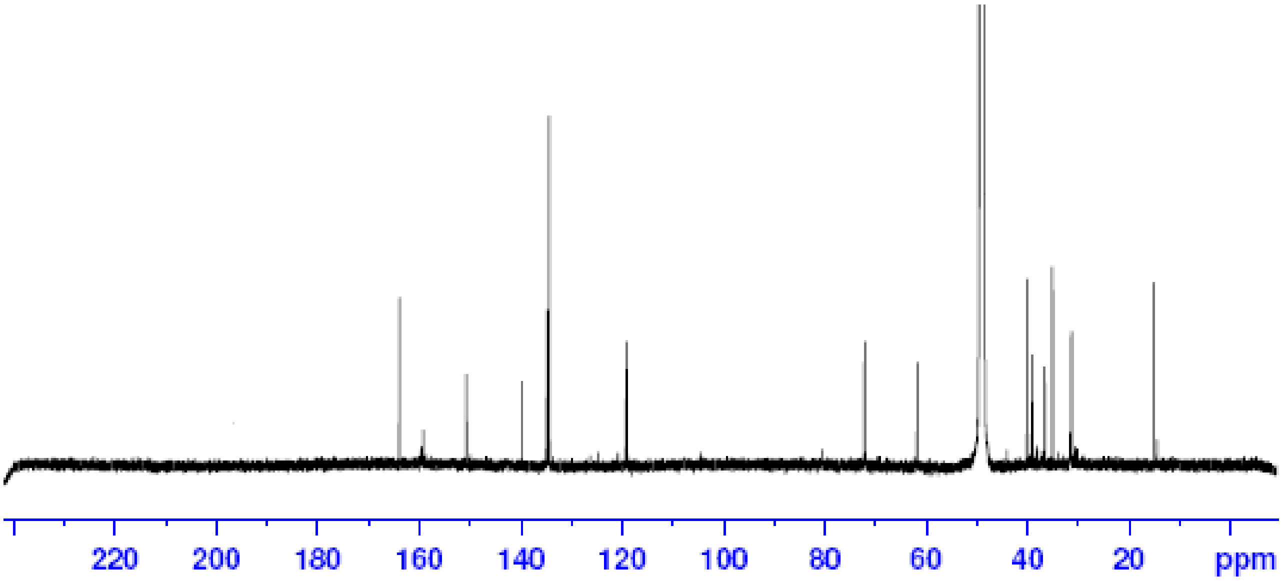

Supplement: Supplementary File 1: — PDF-Document (PDF, 438 KB) [file marinedrugs-10-02492-s001.pdf]
